# The Role of Postbiotics in Asthma Treatment

**DOI:** 10.3390/microorganisms12081642

**Published:** 2024-08-11

**Authors:** Konstancja Węgrzyn, Agnieszka Jasińska, Kamil Janeczek, Wojciech Feleszko

**Affiliations:** 1 Central Clinical Hospital, Medical University of Warsaw, 02-097 Warsaw, Poland; 2 Department of Pediatric Pneumonology and Allergy, University Clinical Centre, Medical University of Warsaw, 02-097 Warsaw, Poland; agnieszka.kaczynska@wum.edu.pl; 3 Department of Paediatric Pulmonology and Rheumatology, Medical University of Lublin, 20-059 Lublin, Poland; kamil.janeczek@umlub.pl

**Keywords:** postbiotics, allergic asthma, microbiome, bacterial lysates, immunomodulation

## Abstract

In recent years, there has been abundant research concerning human microbiome and its impact on the host’s health. Studies have shown that not only the commensal bacteria itself, but also postbiotics, understood as inanimate microorganisms, possibly with the presence of their components, may themselves have an effect on various elements of human physiology. In this review, we take a closer look at the specific ways in which postbiotics can alter immune response in allergic asthma, which is one of the most prevalent allergic diseases in today’s world and a serious subject of concern. Through altering patients’ immune response, not only to allergens but also to pathogens, postbiotics could have a significant role in lowering the number of asthma exacerbations. We suggest that more profound research should be undertaken in order to launch postbiotics into clinical standards of asthma treatment, given the greatly promising findings in terms of their immunomodulating potential.

## 1. Introduction

**Figure 1:**
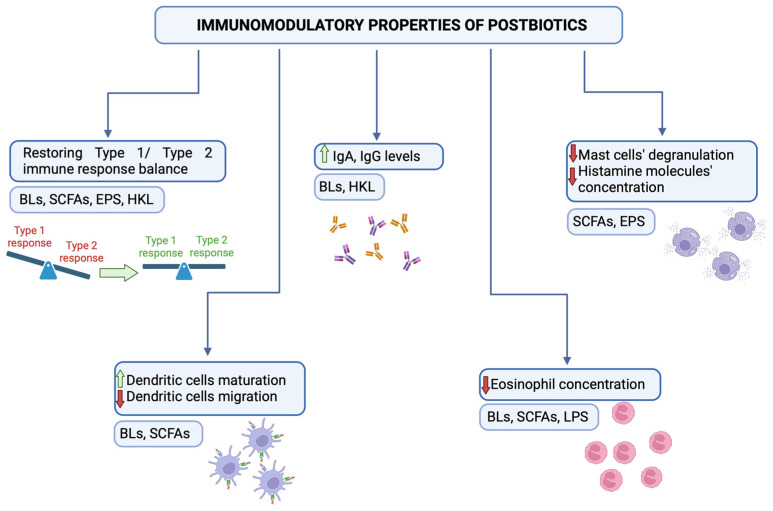
Immunomodulatory properties of postbiotics. Postbiotics act as immunomodulators and promote antiallergic immune responses. They provide the maintenance of violated Th1/Th2 type immune response balance, stimulate IgA and IgG production, and provoke DC maturation. Moreover, they reduce asthma symptoms by decreasing mast cell degranulation and eosinophil count. Created with biorender.com. HKL—Heat-Killed *Lactobacillus*.

In the 21st century, research on microbial compounds and their favorable impact on immune system has gathered pace, as it is now presumed to be one of the crucial factors that stimulate immune system development [1]. This is the reason why the use of probiotics and prebiotics has attracted recent interest. Probiotics are live bacteria strains that present health benefits for the host, compete with pathogens, promote microbial antagonism, and inhibit bacterial toxin production [2]. Prebiotics as specific dietary ingredients affect gut microbiota composition and support probiotics [3]. Strikingly, in many cases, the beneficial role of probiotics results from the effects of postbiotics (which are inanimate microorganisms) and/or their components that confer a health benefit on the host [4]. Postbiotics’ advantages over probiotics include aspects such as the absence of bacterial translocation and a lesser risk of infection deterioration. Moreover, they promote immune system development, inhibit inflammation, prevent infections, regulate lipid metabolism, and stabilize gut microbiota composition [5]. Among them, the most common are bacterial lysates (BLs), short-chained fatty acids (SCFAs), exopolysaccharides (EPSs), and heat-killed *Lactobacilli* [6]. Although SCFAs and EPSs cannot be classified as postbiotics in and of themselves, they are still to be found in the presence of bacterial biomass and therefore present beneficial outcomes for the host [4]. For this reason, we decided to discuss their great immunomodulatory potential in atopic asthma within this article (Figure 1).

Asthma, one of the most common of allergic diseases, affecting approximately 14% of children and young adults worldwide, is a disease with strikingly prominent diversity of ontogenetic and environmental factors modulating its course [7,8]. At its core lie the innate immune system’s acts of recognizing and processing internal and external stimuli, leading to various different ways in which the adaptive response is triggered [9]. Previously, asthma was considered to be a single diagnosis with standardized treatment for all patients. However, it is now known as a heterogenous, multifactorial disorder, and a new approach acknowledging the modulation of the immune response with various specific molecules allows researchers to broaden the possibilities in asthma treatment, focusing on the immunomodulatory properties of postbiotics. This publication aims to summarize and explain the immunomodulatory effects of postbiotics, SCFAs, and EPS in the prevention of exacerbations and the treatment of allergic asthma.

## 2. Methods of Acquiring Data

**Figure 2:**
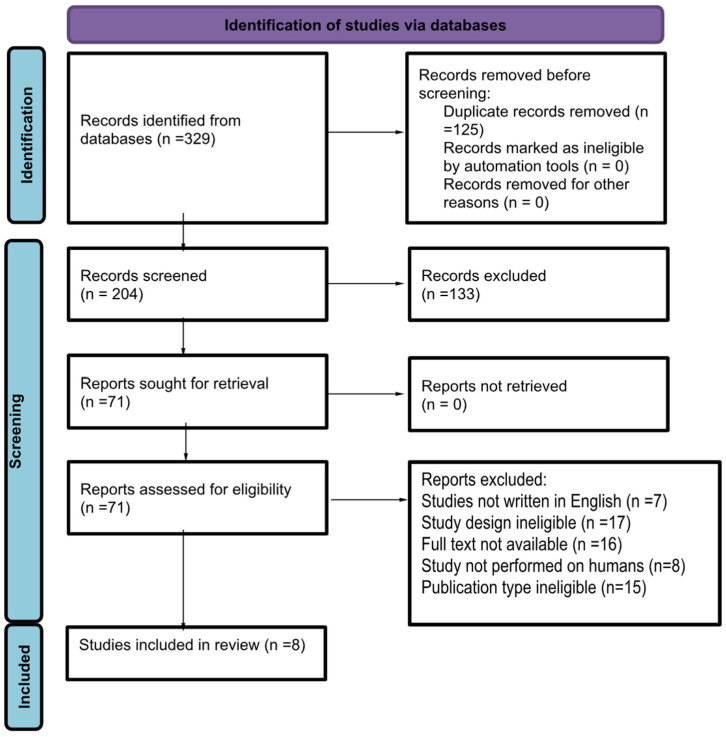
Identification of studies via databases.

The authors searched biomedical databases (PubMed, Scopus, and Web of Science) for articles concerning the use of postbiotics in the asthma treatment. In all databases, the following keywords were searched: “asthma”, “postbiotics”, “bacterial lysates”, “short-chained fatty acids”, “exopolysaccharides”, and “heat-killed *Lactobacillus*”. The research was performed by two independent persons. All the relevant studies were identified by title and abstract reading. Duplicated articles were initially excluded. A careful analysis of full texts was carried out (Figure 2).

In this narrative review, the section focusing on the use of BLs took the form of a systematic review. The authors decided on this approach because BLs represent a well-defined and extensively studied category of postbiotics, warranting a more rigorous and structured analysis. A systematic review methodology ensures a comprehensive and unbiased synthesis of existing evidence, allowing for a more precise evaluation of the efficacy and safety of BLs in asthma treatment. In contrast, the authors chose a narrative approach for other postbiotics due to the lack of available studies on humans. The absence of human studies makes it difficult to apply the same systematic review approach, necessitating a broader, narrative discussion of their potential benefits and theoretical underpinnings.

Studies to be included in the section regarding BLs had to meet all of the following inclusion criteria: clinical trial (double-blind RCT or open-label RCT or sequential trial or cohort study); study on humans (children or/and adults) with asthma; BLs (PMBL or PCBL) as an intervention (alone or combined with standard care); control group receiving only standard asthma treatment or placebo or both. All studies had to be written in English. Study size was not included as a criterion. Studies were excluded if the study design was ineligible, if they included tests performed on animals, or if they evaluated the use of BLs for asthma prevention. 

Two authors independently extracted the following data from the eligible studies: study design, sample size, participants’ characteristics, interventions, comparators, and clinical and other outcomes.

## 3. Bacterial Lysates

BLs are immunomodulatory preparations consisting of antigens derived from the most common respiratory tract bacteria species [10]. They can be obtained with chemical or mechanical lysis—polyvalent mechanical bacterial lysate (PMBL) or polyvalent chemical bacterial lysate (PCBL), which indicates their different biological effects [11]. BLs can be administered orally, sublingually, or intranasally in various forms [12]. Each route of administration provides slightly different effects; thus, it should be chosen considering the type of allergic disease and patients’ preferences [13]. Patients with asthma usually receive the oral form, which mainly affects mucosal immune cells in the intestine [14]. 

BLs’ fundamental mechanism of action is based on the natural immune response provoked by pathogens. Bacterial antigens continuously stimulate the lymphoid tissue in the mucosa to produce cytokines that modulate the immune response, both locally and generally [15]. These immunological changes provide a beneficial switch in the cytokines’ classes and affect the clinical course of the disease [16]. 

Toll-like receptors (TLRs) expressed on dendritic cells (DCs) and monocytes are the first-line proteins that recognize and respond to antigens. They stimulate DCs to maturate and influence cytokine release [17]. Studies suggest that BLs’ activity mainly depends on TLR2 and TLR4 signaling which suppresses airway hyperreactivity, mucus production, and Th2-type immune response in the lungs and lowers the risk of asthma development [18,19,20]. BLs promote the production of IL-10, IL-12, and IFN-gamma characteristic of Th1-type immune response [21,22] and suppress the secretion of Th2-type cytokines IL-4, IL-5, IL-13, and IL-17, which maintains the disturbed Th1/Th2 balance [23,24,25]. Sublingually administered BLs increase the level of NK cells in asthmatic children [24,26]. They are also known to activate peripheral blood macrophages and increase serum levels of IgA, IgG, and human beta-defensin 1 (hBD-1) [27,28]. Furthermore, they seem effective in lowering eosinophil counts in bronchoalveolar fluid and blood [29]. BLs’ mechanism of action was described in depth in our previous publication [30].

Nine human studies evaluated the efficacy of BLs in the prevention of exacerbations and the treatment of asthma. The very first clinical trial was conducted in 1987 by Weiss et al., who investigated the impact of Broncho-Vaxom (PCBL) on patients with asthma or chronic obstructive pulmonary disease (COPD). The research showed that PCBL reduces IgE and increases IgG levels in atopic patients. Although the results did not attain statistical significance, they pointed out the possible beneficial effects of BLs and encouraged scientists to conduct further research [31]. The effectiveness of BLs in reducing asthma exacerbations and symptom severity was confirmed in five clinical trials [23,32,33,34,35]. Both PCBLs and PMBLs were more effective in facilitating the clinical course of the disease than standardized care (SC) [23,33,34]. Only the study by de Boer et al. did not show any difference in the number of exacerbations in the PCBL and SC groups. However, FEV1 was increased in the PCBL group; thus, some improvements were observed [21]. On the other hand, Roßberg et al., who evaluated the effect of BLs administered in infancy on the risk of developing atopic dermatitis (AD), allergic rhinitis (AR), and asthma, concluded that they do not reduce the risk of allergic diseases. Nevertheless, patients received a mixture of heat-killed *Escherichia coli* and *Enterococcus faecalis*; thus, the effects probably resulted from the specific antigen properties and the results should not be clinching [36]. Therefore, further research on the efficacy of BLs in asthma prevention should be conducted (Table 1).

## 4. Short-Chained Fatty Acids

Short-chained fatty acids (SCFAs) are found in the human gut as a product of the anaerobic fermentation of non-digestible dietary fiber and amino acids by saprophytic bacteria, as well as being, in marginal amounts, derived directly from the diet [37]. Although the most abundant SCFA in the human gut tends to be acetate, the most beneficial in regard to health are found to be propionate and butyrate, produced mostly by *Bacteroidetes* and *Firmicutes*, respectively [38,39].

SCFAs can be either absorbed by the gut lining endothelium cells to serve as an energy source or enter the bloodstream and modulate the immune response [37]. Said immunomodulating effects are enabled via transporters, namely, proton-coupled monocarboxylate transporter isoform 1 (MCT1) and the Na+-coupled monocarboxylate transporter 1 (SMCT1), which can be found on the apical surface of colonocytes as well as on the surface of immune system cells [40,41]. Along with the MCT1 and SMCT2 transporters, SCFA uptake into the immune system cells is also facilitated by the direct activation of butyrate-sensing G protein-coupled receptor (GPCR) class, namely, GPR41/FFAR3 (free fatty acid receptor 3), GPR43/FFAR2, and GPR109A/HCAR2, as well as the activation of peroxisome proliferator-activated receptors (PPARs), which promote the influx of SCFAs into the immune cells, and butyrate-sensitive histone deacetylases (HDACs). The essence of SCFA’s immunomodulation potency lies in the deacetylation of histone complexes’ lysine. As a result, chromatin formation becomes denser and firmer so that gene expression becomes suppressed, inhibiting the immune system cells’ natural functions [37]. In various studies, the knockout of GPR41 and GPR43 has been proven to exacerbate asthma responses in mice, leading to assumptions that SCFAs can have a substantial effect on soothing asthmatic symptoms [42]. In a recent systematic review regarding the effects of SCFAs on allergic diseases in humans, a protective effect of higher SCFA levels was shown against allergic diseases, including asthma [43]. There is, however, still a need for further research in this area.

Th2 cell induction was shown to be suppressed in mice with high SCFA levels [44]. Moreover, DCs were proven to polarize naïve CD4+ T cells away from type 2 maturation and instead lean toward type 1 maturation [45]. Furthermore, in the in vitro model, SCFAs decreased DCs’ migration abilities, resulting in decreased asthmatic symptoms [46]. Another relevant element of the immunological pathway in allergy response are regulatory T lymphocytes (Tregs). Tregs have the ability to suppress the inflammation response, regulate the Th1/Th2 imbalance, and promote the remodeling of the airways via the secretion of IL-10 and transforming growth factor beta (TGF-β). What is more, Tregs can derive from naïve CD4+ T cells in the presence of TGF-β [47]. SCFA levels have been proven to positively correlate with the abundance of Treg cells. They have also been found to play a crucial role in Treg cells’ differentiation not only in mice [48,49] but also in humans [50]. 

Kim et al. showed in a study on AD that in mice individuals with higher SCFA levels, the eosinophilic percentage and eosinophilic count were decreased [51]. Given the atopic nature of asthma and AD, this correlation of high SCFA level to low eosinophils seems promising in discovering new therapeutic possibilities in asthma. In other studies, butyrate has been revealed to induce eosinophil apoptosis and reduce their adhesion to endothelial cells. The above-mentioned Zn2+-dependent class I, II, and IV HDACs have also been proven to inhibit eosinophilic survival and migration after exposure to propionate and butyrate [52]. In this light, SCFAs might contribute to alleviating symptoms of atopic asthma.

In allergic response, after being stimulated by IL-4 and IL-13, B cells mature into IgE, producing plasma cells. This ability of maturation and class-switching has been reported to be lower in mice with a high-fiber diet through epigenetic alterations [53]. Propionate and butyrate also decreased IL-4 levels, which is essential to differentiate B cells into IgE-producing cells [54]. Moreover, SCFAs were shown to inhibit mast cell degranulation and release airway contractiveness [55].

Another element of allergic diseases, including asthma, is the dysfunction of the airway epithelium barrier. SCFAs have been discovered to enhance the epithelial wall barrier function [56]. 

The imbalance of gut microbiota is a profound factor contributing to immunological diseases. It has been shown that amongst children suffering from asthma, the levels of SCFAs were significantly lower than in their healthy peers [57]. 

## 5. Exopolysaccharides

EPSs are carbohydrate polymers forming the external coating of the bacterial cell wall. They have diverse health effects, such as calcium and magnesium absorption, glycemic control, and anticarcinogenic and antioxidant effects [58]. Notably, EPSs produced by commensal bacteria, like *Lactobacillus* or *Bifidobacterium*, present immunomodulatory properties [59]. Schiavi et al. revealed that EPSs derived from *Bifidobacterium longum* inhibit eosinophilic migration to the airways, which was connected to Th2-associated interleukin IL-4 and IL-13 decrease [60]. A recent study showed that EPSs isolated from *Bacillus subtilis* divested asthmatic inflammation, linked to the concentration-dependent decrease in IL-4 and IL-5 levels, leading to reduced eosinophilic count [61]. Moreover, EPSs strongly bind the histamine molecules to the surface of bacterial wall and lower their blood count, mitigating the asthmatic response [62]. In a randomized, double-blind, placebo-controlled clinical trial where patients with airborne allergy were administered EPSs derived from *Lactobacillus paracasei* for 12 weeks, there was a significant alleviation of allergic symptoms reported by patients, which also correlated with the decrease in biochemical signs of allergic inflammation [63].

Knowing the effect of EPSs on various elements of the immune system and promising results on inhalant allergy in humans, those studies shine a new light on the potential use of EPSs, amongst other postbiotics, in controlling atopic asthma symptoms.

## 6. Heat-Killed *Lactobacillus*

The genus *Lactobacillus* is represented by almost 250 species of Gram-positive, anaerobic bacteria colonizing multiple, diverse habitats, which can be used in many industrial and healthcare applications [64]. For years, beneficial effects of *Lactobacillus* spp. were observed in preventing gut dysbiosis and allergy development [65,66]. Additionally, a recent airway microbiome profiling revealed the presence of *Lactobacillus* spp. in the nasopharynx and pointed out its remarkable role in local immune changes and the inhibition of respiratory tract pathogens’ growth and virulence [67]. However, there are still insufficient data establishing whether the above-mentioned abilities result from the activity of live bacteria or bacterial products and particles that influence the host’s immunity. 

In an allergy model, mice fed with heat-killed *L. casei* showed significantly lower IgE and IgG1 levels and suppressed T cell production of Th2 type (IL-4, IL-5, IL-10, IL-13) and proinflammatory (IFN-γ and TNF-α) cytokines compared with the placebo group. Moreover, the histological evidence showed the attenuation of lung inflammation and reduced proinflammatory cytokines in bronchoalveolar fluid [68]. Choi et al. observed similar phenomena when examining the impact of heat-killed *Lactobacillus* spp. on dust-mite-induced AD in mice. Moreover, the above-mentioned supplementation ameliorated symptoms and reduced the number of mast cells and eosinophils in lesions. Among 18 *Lactobacillus* strains, *L. brevis* NS1401 induced the greatest IFN-γ and IL-12 secretion and the least IL-4 production [69]. This suggests that the immunomodulatory abilities depend on the *Lactobacilli* species and are unequal for the whole genus. Hong et al. compared the effects of three heat-killed *Lactobacilli* on airway hyper-responsiveness in a murine asthma model. The research showed that airway inflammation was suppressed in *L. plantarum*- and *L. curvatus*-treated mice, and lower IL-4 and IL-5 levels were observed. On the contrary, in the *L. sakei* subsp. *sakei*. group, no differences were found [70]. Similar results were shown by Lee et al., who compared cytokine regulatory effects of three *Lactobacilli* strains in an in vitro study. Notably, *Lactobacilli* lysates were more likely to stimulate cytokine production than heat-killed bacteria, cell supernatants, and live strains. Lipoteichoic acid isolated from bacteria promoted TNF-alpha production via TLR2-mediated NF-κB and extracellular-signal-regulated kinase (ERK) activation [71]. Heat-killed *Lactobacilli* strains stimulate DCs to produce IL-12 p70 and switch T cells to Th1 type immune response [72]. Moreover, they suppress the production of IL-6 and IL-17A, which results in Treg/Th17 balance maintenance [73]. The above-mentioned studies suggest that tyndallization does not reduce the immunomodulatory abilities *of Lactobacillus spp.* and the results of their use are comparable with those from live bacteria. Furthermore, heat-killed bacteria offer increased safety; thus, their usage in clinical practice should be considered.

Currently, there are insufficient data concerning their use in asthmatic patients. Nevertheless, promising effects of tyndallized *Lactobacillus* spp. are shown in research on other allergic diseases; thus, maybe in the future, extensive sample studies will be performed [74].

## 7. Conclusions

As described above, postbiotics seem to be promising therapeutic approaches in asthma treatment. They undoubtedly affect the immune system and promote various changes in cytokine production, T-cell differentiation, immunoglobulin release, and eosinophil infiltration. Lowering the number of asthma exacerbations as the effect of BLs’ immunomodulating qualities could insinuate their appropriate use in asthma treatment. However, their underlying mechanisms of action are not fully elucidated, and it is difficult to determine whether they should be applied in clinical practice. Moreover, postbiotics, excluding BLs, have not been studied in human subjects with asthma, making it challenging to determine their efficacy in treatment. Nevertheless, existing research indicates that their immunomodulatory properties could be promising. Thus, further, large-sample studies should be carried out. Health benefits, side effects, and cost-effectiveness should be examined.

## Figures and Tables

**Table 1:** Characteristics of included studies.

Author, Year	Study Design	Subject (BLs/Control)	Mean Age	Treatment Regimen	Clinical Outcomes	Immunological and Other Outcomes
Emeryk et al. 2018 [32]	RCT	150 (74/76)	6–16 years	Ismigen (PMBL) vs. Placebo	The number of asthma exacerbations was lower in the PMBL group.	
de Boer et al. 2021 [21]	RCT	75 (38/37)	16–60 years	OM-85 (PCBL) vs. SC	Exacerbations were not different between groups after 18 months.	FEV1 increased in the PCBL group.
Lu et al. 2015 [28]	RCT	60 (24/36)	5–15 years	OM-85 (PCBL) vs. SC		Increased serum IFN-γ/IL-4 ratio was observed.
Li et al. 2022 [33]	Retrospective PS-matched cohort study	795 (337/458)	6 months–14 years	QIPIAN (PMBL) vs. SC	Fewer exacerbations were observed in the PMBL group.	
Bartkowiak-Emeryk et al. 2021 [26]	RCT	49 (21/28)	6–15 years	Ismigen (PMBL) vs. placebo		Increased serum T lymphocyte, CD4+ CD25+ FOXP3+, CD8+, CD3− CD16+ CD56+. Decreased serum CD69+ and CD25+ subset of CD3+.
Koatz et al. 2016 [34]	open-label, prospective, sequential	28	16–65 years	1st year SC; 2nd year OM-85 (PCBL)	Decreased symptom severity and the number of exacerbations.	Increased serum and salivary secretory IgA.
Han et al. 2016 [23]	RCT	136 (74/62)	7 months–5 years	OM-85 (PCBL) vs. inhaled corticosteroids/aminophylline/antibiotics	Decreased the frequency and duration of capillary bronchitis and asthma.	Decreased serum IL-4 and IL-17 levels. Increased serum IL-10 and IFN-g levels.
Abdou et al. 1993 [35]	RCT	50(25/25)	Not applicable	OM-85 vs. SC	Reduced the duration and number of asthma attacks.	Increased FEV1/FVC% ratio. Increased serum IgA, IgM, and IgG levels. Decreased serum IgE level. Decreased eosinophil count in bronchoalveolar fluid. Increased IgA/albumin ratio.

RCT—randomized controlled trial; PMBL—polyvalent mechanical bacterial lysate; PCBL—polyvalent chemical bacterial lysate; SC—standard care; FEV1—forced expiratory volume during the first second of expiration.

## Data Availability

No new data were created or analyzed in this study. Data sharing is not applicable to this article.
